# Helping the Released Guardian: Drug Combinations for Supporting the Anticancer Activity of HDM2 (MDM2) Antagonists

**DOI:** 10.3390/cancers11071014

**Published:** 2019-07-19

**Authors:** Justyna Kocik, Monika Machula, Aneta Wisniewska, Ewa Surmiak, Tad A. Holak, Lukasz Skalniak

**Affiliations:** Department of Organic Chemistry, Faculty of Chemistry, Jagiellonian University, Gronostajowa 2, 30–387 Krakow, Poland

**Keywords:** HDM2 antagonist, MDM2, p53, p21, synergy, combined cancer treatment, targeted therapy, chemotherapy, nutlin, RG7388, idasanutlin

## Abstract

The protein p53, known as the “Guardian of the Genome”, plays an important role in maintaining DNA integrity, providing protection against cancer-promoting mutations. Dysfunction of p53 is observed in almost every cancer, with 50% of cases bearing loss-of-function mutations/deletions in the *TP53* gene. In the remaining 50% of cases the overexpression of HDM2 (mouse double minute 2, human homolog) protein, which is a natural inhibitor of p53, is the most common way of keeping p53 inactive. Disruption of HDM2-p53 interaction with the use of HDM2 antagonists leads to the release of p53 and expression of its target genes, engaged in the induction of cell cycle arrest, DNA repair, senescence, and apoptosis. The induction of apoptosis, however, is restricted to only a handful of p53^wt^ cells, and, generally, cancer cells treated with HDM2 antagonists are not efficiently eliminated. For this reason, HDM2 antagonists were tested in combinations with multiple other therapeutics in a search for synergy that would enhance the cancer eradication. This manuscript aims at reviewing the recent progress in developing strategies of combined cancer treatment with the use of HDM2 antagonists.

## 1. Introduction

The protein p53 is a powerful regulator of cell fate. Together with a complex net of co-regulatory proteins it integrates multiple stress signals and translates them into several programs, such as cell cycle arrest, DNA-repair, senescence or apoptosis. One of the major roles fulfilled by p53 is the protection against early cancerogenic events by the induction of repair mechanisms in response to DNA mutations and by the controlled elimination of extensively damaged cells. For this reason, p53 protein has been called the “Guardian of the Genome”. Not surprisingly, p53 is found functionally impaired in almost every cancer type. Therefore, since the discovery of its tumor-suppressive potential, the strategies of restoring its activity in cancer cells has become the Holy Grail of modern medicinal chemistry.

The progress in this field is best reflected by the development of small molecule antagonists of HDM2 (mouse double minute 2, human homolog) protein. HDM2 (in mouse known as Mdm2) is an E3 ubiquitin ligase that binds p53, blocks its trans-activating activity, governs its nuclear export and targets p53 to proteasomal degradation. While deactivation of p53 is a virtually obligatory step in cancer development [1], it is estimated that around 50% of cancers contain non-mutated (wild type) *TP53* genes, which encode a functional p53 protein. In those cells, however, the activity of p53 is blocked predominantly by the overexpression of HDM2 protein, frequently achieved by the duplication of the *HDM2* gene. In such cells, the administration of HDM2 antagonists results in forced dissociation of HDM2-p53 complexes, releasing p53 from HDM2 inhibition [2]. Such a forced p53 release results in the expression of a plethora of p53-regulated genes, presenting a transcriptome landscape similar, but not identical to that of genotoxic p53 activation [3]. However, the blockade of HDM2 protein leads only to partial activation of p53, and thus the outcome of such activation differs from the full p53 activation process observed in response to genotoxic stress.

## 2. Limited Elimination of Cancer Cells by HDM2 Antagonists

It has been well documented that the activation of p53 by HDM2 antagonists results in the inhibition of the growth of p53^wt^ cancer cells, both in vitro and in mouse xenograft models. However, while initially it has been expected that the activated p53 would lead to strong apoptosis in developed p53^wt^ cancers, with time, growing evidence pointed to serious limitations of this treatment strategy. First, it soon became clear that HDM2 antagonists induce apoptosis only in a limited subset of p53^wt^ cells [4,5]. In many additional p53^wt^ cell lines HDM2 antagonists induce cell cycle arrest, which is more like reversible quiescence rather than irreversible senescence [6]. Although the growth inhibition of cancer cells provides significant short-term therapeutic effects, the limited elimination of cancer cells gives them the time necessary to gain new genetic or epigenetic features that lead to the generation of secondary resistance. Such a phenomenon has been reported for almost every significant HDM2 antagonist [7,8,9,10,11,12,13,14,15]. Additionally, a recent study performed with the use of 113 p53^wt^ cell lines showed that 70 of them are naturally resistant to such a treatment [16].

The limited apoptosis upon p53 release by HDM2 antagonists likely results from distinctive modes of p53 activity that leads either to cell cycle arrest and DNA repair or cell death. In the first mode, the activation of p53 results in the induction of the expression of proteins engaged in cell cycle arrest, such as p21 [17], and the proteins that assure negative feedback loops required for the generation of p53 activation pulses, such as HDM2 and Wip-1 (wild-type p53-induced phosphatase 1) [18,19]. Cell cycle arrest is also partially related to the transcriptional downregulation of genes related to the cell cycle. In this process, DREAM (dimerization partner, RB-like, E2F and multi-vulval class B) protein complex acts as a transcriptional repressor by binding to E2F and CHR (cell cycle genes homology region) elements. This p53-p21-DREAM pathway regulates the expression of over 250 genes, most of which are involved in the cell cycle. Moreover, DREAM complex controls genes responsible for DNA repair, telomere maintenance and chromosomal instability [20]. Given the nature of p53 in this mode, it is called p53_ARRESTER_ and is characterized by the phosphorylation at Ser15 and Ser20 residues [21]. The second mode requires additional phosphorylation of p53 at the Ser46 residue, resulting in a so-called p53_KILLER_ [22]. This version of p53 results in the induction of apoptosis at least partially by the activation of a positive p53-PTEN (phosphatase and tensin homolog deleted on chromosome ten)-Akt-HDM2 loop [23] and induction of the expression of pro-apoptotic Bax protein [24]. Such a complex but fine regulation of p53 activity provides a tunable, coordinated response to DNA damage, first giving the cell a chance to fix the damage, but eliminating the cell if the repair machinery should have failed.

**Figure 1:**
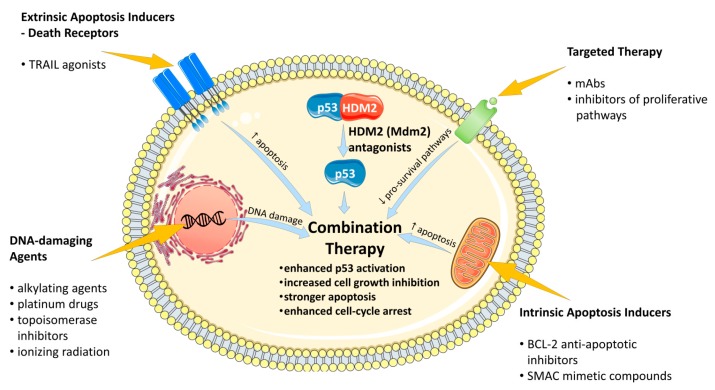
The outline of the main strategies of supporting the action of HDM2 (mouse double minute2, Mdm2, human homolog) antagonists in the recent experimental combination therapies.

Since the transition from p53_ARRESTER_ to p53_KILLER_ requires the accumulation of HIPK2 kinase (homeodomain-interacting protein kinase 2) in response to severe DNA damage [25] and normally occurs after several cycles of the induction of p53_ARRESTER_ protein [22,26,27], it is not easily achieved by a rough p53 release evoked by HDM2 antagonists. Additionally, the forced release of p53 by HDM2 antagonists seem to be devoid of the proper cellular context and other mechanistic features, that is required for enabling proapoptotic functioning of p53 [28]. Moreover, it was proposed that it is the pulse of p53 activation in the G2 phase of the cell cycle which result in irreversible senescence of DNA-damaged cell, while HDM2 antagonists arrest the cells predominantly in the G1 phase, where the cells remain the potency of re-entering mitosis [29]. For these reasons, HDM2 antagonists require the use of supporting agents that would provide synergistic therapeutic effects. In fact, besides a substantial work on the optimization of lead molecules [30,31], much effort has been done in the last ten years in the field of the development of combined treatment strategies, that would incorporate HDM2 antagonists into successful anti-cancer treatment regimes. The successful combinations attempt to support HDM2 antagonists most often by inducing natural DNA damage signaling (achieved with DNA-damaging chemical agents or irradiation), blocking pro-survival or p53-inhibitory circuits (inhibitors of signaling kinases, such as Akt, (protein kinase B) or phosphatases, such as Wip-1), or increasing proapoptotic signaling in cancer cells (inhibitors of antiapoptotic proteins, TRAIL (TNF-related apoptosis-inducing ligand) agonists etc.) (Figure 1).

In this manuscript, we first shortly outline the most important and most potent HDM2 antagonists, and then review the potential positive therapeutic interactions of these molecules with well-established anti-cancer agents, grouped by the general mechanism of action of these agents (see also Table 1).

## 3. The Most Significant Representatives of HDM2 Antagonists

**Figure 2:**
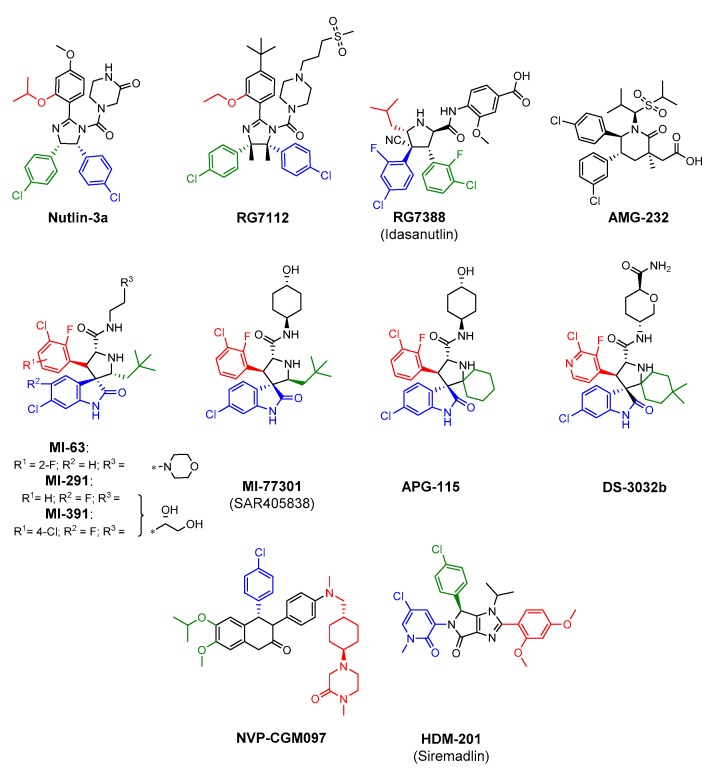
Structures of the potent HDM2 antagonists used in combination therapies. Binding of the compounds with protein HDM2 subpockets based on crystal structure or predicted is color labeled: Trp23—blue; Phe19—red; Leu26—green.

Since the discovery of the first potent small-molecular inhibitors of HDM2-p53 interactions, numerous chemical scaffolds have been published and tested [31,109]. Several promising compounds are currently being tested in cancer-related clinical trials [30,110]. In general, these formulas are designed using the three-finger pharmacophore model in which three substituents are selected to mimic natural interaction between p53 and HDM2 proteins based on Phe19, Trp23 and Leu27 amino acids of the p53 side chain (Figure 2) [111,112]. 

One of the earliest HDM2 antagonists, discovered by Hoffman-La Roche (Basel, Switzerland) in the early 21st century, are called the nutlins and are built around the *cis*-imidazoline core [113]. Following the initial discovery of **nutlin-3a** in 2004, the more potent representative of this family, **RG7112**, was developed [114]. The co-crystal structures with HDM2 (for example see PDB ID: 4HG7, 4JRG) showed that the two *cis*-oriented phenyl rings occupy Leu27 and Trp23 pockets, whereas the alkoxy group (isopropoxy in case of **nutlin-3a** and ethoxy in **RG7112**) fits into Phe19 pocket. Further research by Roche yielded the second generation compound **RG7388** (**idasanutlin**), which is built on the pyrrolidine core [115]. The main optimization step, besides the core, was a change of the mutual configuration of the phenyl rings from *cis* to *trans*, which resulted in its reversed occupation in the HDM2 subpockets. Further, to reduce exposure variability and increase the safety and efficiency of **idasanutlin**, its PEGylated form was developed and tested (**RG7775**). Another similar inhibitor, **AMG-232** was discovered by Amgen Inc. (Thousand Oaks, California, USA) as a result of rational design and optimization steps of the initial lead structure [76]. The idea which lies behind the piperidinone scaffold is similar to the ones of nutlins. The isopropyl substituent fills the Leu26 pocket, whereas two *trans*-orientated chlorophenyl groups occupy the Trp23 and Phe19 pockets (PDB ID: 40AS).

Another numerous class of HDM2 antagonists are spiro-oxindole-3,3′-pyrrolidines, first discovered by Ding et al. at the University of Michigan [116]. Further research yielded the compound **MI-63** and closely related **MI-219** and **MI-319** [117,118]. The analysis of the co-crystal structures of **MI-63** analogs suggests that the oxindole part tightly occupies the Trp23 pocket, the aliphatic neo-pentyl substituent fits into Phe19 pocket, and substituted phenyl fits into the Leu26 pocket (for example see PDB ID: 3LBL). Further optimization of spiro-oxindoles led to the discovery of second-generation compounds with reversed stereochemistry and increased affinity [119]. The co-crystal structure of **MI-77301** with HDM2 reveals the binding mode similar to its precursors, though it creates additional interactions and induces refolding of the unstructured N-terminal region of HDM2 (PDB ID: 5TRF). Last years provided a group of third-generation spiro-oxindoles, based on dispiropyrrolidine core. The most potent representatives are **APG-115** developed by Ascentage Pharma Group (Suzhou, China) [120] and **DS-3032b** by Daiichi-Sankyo (Tokyo, Japan) [121].

Two additional classes of HDM2 antagonists, being currently tested in clinical trials, were developed by Novartis (Basel, Switzerland). The design is based on the new, ‘central valine’ concept, which reflects the idea of placing a planar-aromatic ring near the Val93 residue [122]. The first design was based on the dihydroisoquinolinone core and led to the highly potent compound **CGM097** [123]. In its co-crystal structure, the molecule fits well into the classical binding pocket on the HDM2 surface (PDB ID: 4ZYF). The Leu26 subpocket is occupied by the alkoxy derivative, the Trp23 subpocket is filled with the 4-chlorophenyl substituent, and the elongated dialkylated aniline locates in the Phe19 subpocket. Further development of the dihydroisoquinoline scaffold resulted in a new class of compounds with the pyrazolo-pyrrolidinone core, represented by the **HDM-201** (siremadlin) compound [124]. The rational design enforced the pseudo-equatorial orientation of its 4-chlorophenyl moiety placed in the binding pocket, and as such fulfilled the concept of the ‘central valine’ model. 

Except for small-molecular inhibitors, there are examples of peptide and peptide-mimetics which can interact with HDM2 protein. Noteworthy, one of them, **ALRN-6924**, discovered by Aileron Therapeutics (Cambridge, Massachusetts, USA), is currently involved in several clinical trials [30]. Its structure is based on the idea of a p53-derived peptide chain, ‘stapled’ in a helical configuration by the hydrocarbon chain. This approach is beneficial because (i) covalent modification is stabilizing the α-helix of the peptide, (ii) the staple moiety protects the peptide from proteolytic degradation, and (iii) cyclization enables the compound to cross the cell membrane. A molecule named as **KRT232** delivered by Kartos Therapeutics (Redwood City, California, USA) is in some clinical trials, though its structure has not been disclosed.

## 4. Drug Combinations for the Supporting of Anti-Cancer Activity of HDM2 Antagonists

### 4.1. Combinations with DNA-Damaging Agents

DNA is the most important target for cancer chemotherapy due to the high division rate of cancer cells and improper functioning of DNA repair mechanisms in those cells. For these reasons, any factor causing DNA mutations, i.e., any genotoxic drug or ionizing radiation, would cause serious, irreversible defects in the cell genome that would be more harmful to cancer cells than normal cells. Since DNA damage itself induces a physiological p53 response, the addition of HDM2 antagonists to DNA-damaged cells may assure a prolonged p53 activation by blocking HDM2-negative regulatory loop. At the same time, DNA-damaging agents provide a complete activation of p53, which cannot be achieved when HDM2 antagonists are used alone. 

**Figure 3:**
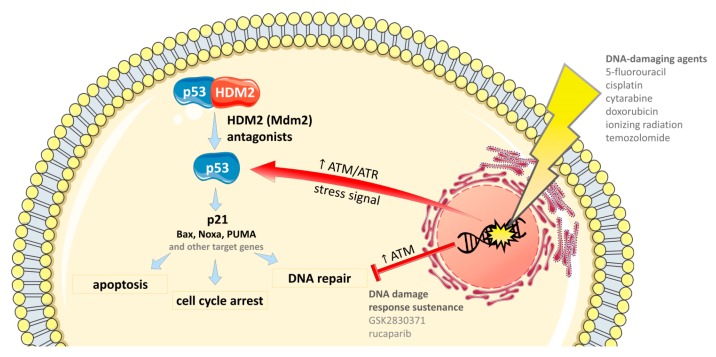
The outline of the combinations of HDM2 antagonists with DNA-damaging agents and the drugs that sustain DNA damage response.

There are different mechanisms by which therapeutic agents induce DNA damage in the targeted cancer cells (Figure 3) [125]. In this section, the combination therapies of p53/HDM2 antagonists with DNA-damaging agents will be presented according to their mechanism of action.

#### 4.1.1. Alkylating Agents

Alkylating agents transfer alkyl-groups to DNA and form covalent bonds with nucleobases. This affects the structure and dynamics of DNA, leading to the blockade of DNA replication and RNA transcription, fragmentation of DNA by hydrolytic events and by malfunctions in the action of repair enzymes, as well as nucleotide mispairing. All these events cause DNA damage response that can potentiate the therapeutic effects of HDM2 antagonists.

There are different groups of alkylating agents [125]. Temozolomide (TMZ), which delivers methyl groups to purine bases of DNA, results in the formation of N7-methylguanine and N3-methyladenine. TMZ was evaluated in combination with **RG7775** (**idasanutlin** prodrug) to treat neuroblastoma [55]. The combined treatment has led to the inhibition of tumor growth, which was correlated with the upregulation of genes involved in apoptosis and signal transduction and downregulation of genes involved in DNA replication, mitosis, cell cycle progression and cell division [55]. In another study, TMZ showed an additive antiproliferative effect with HDM2 antagonist **CGM097** in neuroendocrine tumor model [32]. Furthermore, **idasanutlin** was combined with another alkylating agent—busulfan, resulting in consolidation during frontline treatment in neuroblastoma cells [34]. Trabectedin, the compound which alkylates guanine at the N2 position, leads to cell-cycle arrest and p53-mediated apoptosis. This compound was combined with **RG7112**, which resulted in significant synergy in liposarcoma cells [56].

#### 4.1.2. Platinum Complexes

Platinum complexes are molecules that form covalent links between DNA nucleobases, bridged by the platinum atom-containing moieties (a mechanism similar to that of alkylating agents) [126]. This forms monofunctional adducts or inter-strand, intra-strand, or DNA-protein crosslinks [127]. As a consequence, DNA bending and inhibition of DNA replication and transcription are observed [125].

Despite many side effects of cisplatin, it was widely evaluated in many combination therapies with p53/HDM2 antagonists. Combination treatment with cisplatin and **nutlin-3a** in sarcoma cell lines revealed a clear synergism. It also reduced the negative genotoxic effects without significant changes in antitumor activity. In nasopharyngeal carcinoma cells (NPC) the same therapy showed that **nutlin-3** made NPC cells more sensitive to cisplatin-related cytotoxicity; another study indicates that non-small cell lung cancer cell lines are more prone to apoptosis after the combination therapy administration [39,40,42]. The treatment with **idasanutlin** and cisplatin in p53^wt^ neuroblastoma cell lines resulted in increased apoptosis, which indicates the synergic effect. In ovarian cancer, the same combination treatment had additive or even synergistic effect, which was confirmed by increased p53 activation, enhanced apoptosis and cell cycle arrest [34,37]. **MI-319** in combination with cisplatin presented increased cell growth inhibition and apoptosis in pancreatic cancer (PC) cells due to the up-regulation of p73 protein, which has some overlapping anticancer activities with the p53 protein [38]. **AMG-232** combined with cisplatin had notably increased antitumor efficacy in many different cancer cell lines and also in the in vivo studies on mice [35].

Successors of cisplatin, carboplatin and oxaliplatin, were introduced to overcome side effects and resistance to cisplatin [128]. Carboplatin is currently tested in combination with **APG-115** in a clinical trial on patients with salivary gland carcinoma (NCT03781986). Carboplatin was also combined with **AMG-232 [35]**, while oxaliplatin was evaluated in combination with **MI-219** on p53^wt^ Capan-2, HCT-116, and MCF-7 cancer cell lines. In all these three types of tumors, the improved growth inhibition was observed [54].

#### 4.1.3. Antimetabolites

Antimetabolites are analogs of naturally occurring compounds that interfere with biochemical processes essential for cell division and survival. Most antimetabolites used in cancer therapy have nucleotide-related structure. These compounds either lead to the inhibition of nucleotide biosynthesis or are incorporated into DNA molecules, leading to impaired DNA processing and mutations [125,129].

Antimetabolites are commonly used to treat cancer and tested also in combination with HDM2 antagonists. Some of the combinations have progressed to the clinical trials. As an example of pyrimidine nucleoside analog, fludarabine (2-fluoro-ara-AMP) has synergic effect with **nutlin-3a** in chronic lymphocytic leukemia (CLL) by increasing p53 levels, inducing Bax conformational changes and apoptosis in p53^wt^ cells [48]. Another antimetabolite, a purine nucleoside analog, cytarabine, is currently under evaluation in combination with **idasanutlin** in patients with relapsed or refractory acute myeloid leukemia (AML) (NCT02545283). This study has recently progressed to phase 3, which makes **idasanutlin** the most advanced HDM2 antagonist in the clinical trials. Cytarabine is also tested in clinical trials with daunorubicin and **idasanutlin** for safety and efficacy determination in newly diagnosed patients with AML (NCT03850535). A study evaluating the combination of **RG7112** with cytarabine in patients with AML is already completed (NCT01635296). Yet another HDM2 antagonist which is evaluated in combination with cytarabine in acute myeloid leukemia or advanced myelodysplastic syndrome patients is stapled peptide **ALRN-6924** (NCT02909972). More pyrimidine analogs were also tested: 5-fluorouracil with **CGM097** [32], gemcitabine with **nutlin-3a** [46] and **MI-63** [49], decitabine with **AMG-232** (NCT03041688), and 5-azacitidine (AZA) with **DS-3032b** (NCT02319369).

#### 4.1.4. Topoisomerase Inhibitors

Topoisomerases (type I and type II) are enzymes that are involved in replication and transcription. Their role is to relax supercoiled DNA. Drugs which target topoisomerases are commonly used to treat cancer [125,130]. Topoisomerase inhibitors do not block the catalytic function of the enzyme, but they “poison” them by weakening the enzyme ability to religate DNA and/or by increasing the DNA cleavage [131].

Topotecan and doxorubicin, which are topoisomerase inhibitors, synergize with **idasanutlin** in p53^wt^ neuroblastoma cells [34]. In another study, irinotecan was combined with **AMG-232** in the colorectal carcinoma model. The combination therapy showed enhanced anti-cancer activity in comparison to monotherapy [35]. Furthermore, a combination of the topoisomerase II inhibitor, etoposide, with **nutlin-3a** led to enhanced activation of effector caspases [46]. Etoposide was also combined with another HDM2 antagonist, **MI-219.** The combinatory treatment revealed that **MI-219** synergizes with etoposide by cytotoxicity enhancement and acceleration of the cell killing process [47]. Doxorubicin was evaluated in combination with **AMG-232** and **nutlin-3a** [33,35,45,46]. Another inhibitor from the same class of drugs, daunorubicin, is under evaluation in clinical trials in combination with **HDM-201** as the first-line treatment of AML patients or in patients with relapsed/refractory AML (NCT03760445). Daunorubicin is also tested in combination with **idasanutlin** in newly diagnosed AML patients (NCT03850535). Another anthracycline—idarubicin— was tested in vitro with **CGM097**. In the experiment that the strongest therapeutic effect was achieved when AML cells lines were treated with both agents [44].

#### 4.1.5. Ionizing Radiation

Ionizing radiation (IR) is widely used in anti-cancer therapy. Many patients during the cancer treatment receive ionizing radiation, that is either applied alone or in combination with anti-cancer drugs [132]. The radiation has a direct or indirect impact on DNA damage [133]. Direct DNA damage is caused by the introduction of DNA strand breaks, while indirect is mediated by the production of reactive oxygen species (ROS), which in turn lead to many DNA modifications like formation of abasic sites, DNA adducts, single-strand breaks (SSBs) and double-strand breaks (DSBs), DNA-protein crosslinks, base oxidation and base deamination [125,134]. Despite the fact that radiation treatment has many side effects [135], it is still a popular approach for the treatment of cancer, especially in combination with therapeutic drugs.

Studies have indicated that IR induces HDM2 expression in a p53-dependent manner. Thus, IR therapies were combined with HDM2 antagonists in an attempt to increase the activity of p53 protein [136]. **Nutlin-3** induces senescence and radiosensitivity of laryngeal carcinoma cells (LSCC) retaining wild-type p53 [52]. In another study, **nutlin-3** radiosensitized lung cancer cells with wild-type p53, resulting in cell cycle arrest and apoptosis after the combined treatment [137]. This combination was also evaluated in human glioblastoma multiforme, where it resulted in increased apoptosis and senescence. Furthermore, **nutlin-3a** strengthened the radiation response of glioma cell lines harboring wild-type p53 [53]. **Idasanutlin** was combined with IR in two models of childhood rhabdomyosarcoma, resulting in increased IR therapeutic efficiency [51]. Another small molecule inhibitor, **APG-115**, was shown to provide the radiosensitization of p53 wild-type gastric cancer cells. The combination of **APG-115** with radiation resulted in cell cycle arrest and apoptosis of these cells [138]. Moreover, **AMG-232** is tested in combination with radiation in clinical trials on the patients with glioblastoma (NCT03107780) and soft tissue sarcoma (NCT03217266). Radiation treatment in combination with **AMG-232** leads to enhanced tumor reduction. Combination of radiation with **AMG-232** was also evaluated in adenoid cystic carcinoma, resulting in tumor growth inhibition and total regression that remained for months after the treatment [50].

### 4.2. Combinations with Drugs that Sustain DNA Damage Response

#### 4.2.1. DNA Repair Inhibitors

Poly(ADP-ribose) polymerase (PARP) is a nuclear enzyme, which function is to repair DNA single-strand breaks. PARP also plays a role in cell proliferation, differentiation, and transformation [139,140]. The pharmacological inhibition of PARP leads to the generation of double-strand breaks, which lead to sustained genotoxic stress and apoptosis (Figure 3). One of the nonclinical studies focused on combining **nutlin-3a** or **idasanutlin** with a PARP inhibitor, rucaparib, in ovarian cancer cell lines [59]. The combination was proved to be effective in p53^wt^ ovarian cancer cell lines, where it induced apoptosis and cell cycle arrest [59].

#### 4.2.2. Phosphatases

Wip1 is a negative regulator of multiple DNA damage response proteins, such as p53, CHK2 (checkpoint kinase 2), histone H2AX, and ATM (Ataxia telangiectasia mutated) (Figure 3) [141]. Overexpression of this protein has been observed in human cancers, particularly of breast and ovarian origin [142]. A Wip1 inhibitor, GSK2830371, potentiated the cytotoxic effect of **nutlin-3**, **idasanutlin**, and **HDM-201**, despite having no cytotoxic activity on its own [15]. The authors explained that the inhibition of Wip1 phosphatase increases the activation of the ATM-mediated network to maintain the phosphorylated state of key proteins engaged in DNA damage response [15]. Another group reports similar observations of **nutlin-3** and GSK2830371, which when applied together increased p53-mediated tumor suppression and induced senescence in MCF7, U2-OS and HCT116 cells [58]. Additionally, **ATSP-7041**, a stapled peptide postulated to act as a dual inhibitor of HDM2 and HDM4, synergized with GSK2830371, triggering p53-mediated cell death in Ewing's sarcoma [57].

### 4.3. Combinations with Apoptosis-Inducing Agents

**Figure 4:**
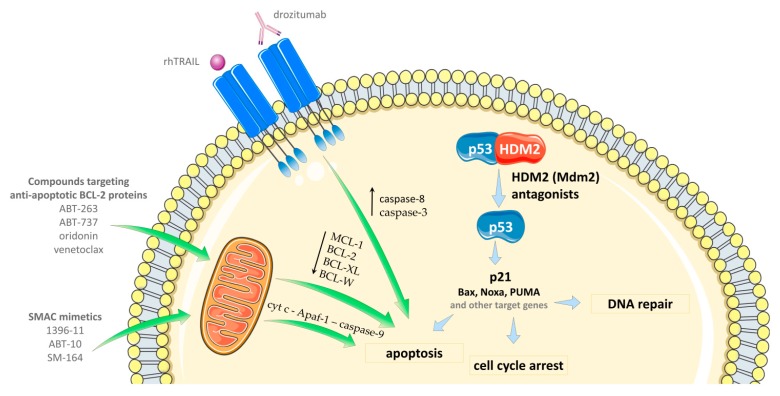
The outline of the combinations of HDM2 antagonists with apoptosis-inducing agents.

The activation of p53 leads to apoptosis due to the disruption of the balance of pro- and anti-apoptotic proteins (directly or per permeabilization of mitochondria’s membrane). This action, however, requires the induction of p53_KILLER_ mode, which mediates the expression of pro-apoptotic proteins, such as Bax, Apaf-1, and Puma [21,143]. HDM2 antagonists are predominantly inducing the p53_ARRESTER_ mode, resulting in insufficient apoptosis. Additionally, the overexpression of anti-apoptotic BCL-2 family proteins (i.e., BCL-2, BCL-X, MCL-1) may inhibit the proapoptotic effect of p53 activation [144,145,146]. Thus, the inactivation/down-regulation of these antiapoptotic proteins or increasing the proapoptotic signaling are rational strategies for enhancing the weak proapoptotic potential of HDM2 antagonists (Figure 4).

#### 4.3.1. Selective BCL-2 Protein Inhibitors

Venetoclax (ABT-199/GDC-0199) is a BH3 (Bcl-2 homolog domain) mimetic, which selectively inhibits BCL-2 protein. Venetoclax has revealed significant anti-tumor activity in both pre-clinical and clinical studies [147,148,149]. The potency of the combined therapy of venetoclax with **idasanutlin** was investigated both in vitro and in vivo on human cancer cell xenograft mice models of AML and neuroblastoma. The venetoclax/**idasanutlin** treatment results in the robust down-regulation of the anti-apoptotic protein MCL-1, which is also associated with the resistance to BH3 mimetics in a single therapy [64,65]. This mechanism was proposed to explain the observed synergy of venetoclax and **idasanutlin** treatment [64]. A phase 1b/2 clinical study with venetoclax in combination with **idasanutlin** or cobimetinib (a MEK inhibitor [150]) for the treatment of AML patients is currently running (NCT02670044). In addition, phase 1b/2 study aims to assess the dose-escalation and regimen of venetoclax and **idasanutlin** in combination with monoclonal antibodies against CD20: obinutuzumab for follicular lymphoma (FL), and rituximab for diffuse large B-cell lymphoma (DLBCL) (NCT03135262).

ABT-737 is a BH3 inhibitor of BCL-2, BCL-XL, and BCL-W proteins [151,152]. The combinations of the compound with nultin-3 and **MI-63** were investigated by Kojima et al., Gu et al. and Carter et al. [60,61,62]. The scientists have confirmed that simultaneous treatment with HDM2 antagonists and BCL-2 inhibitors results in greater induction of apoptosis and reduction of cell viability of AML cells, multiple myeloma, and blast crisis myeloid leukemia cells. Moreover, it has been proved that the synergistic effect of the combination therapy is independent of the cell cycle, even though the cytotoxicity of each agent alone depends on the cell cycle’s phase [60,61,62]. In another study, performed with acute lymphoblastic leukemia (ALL) and AML patients-derived cell lines, the sublines resistant to BCL-2 inhibitors (ABT-737 and its orally active analog ABT-263—navitoclax) or MDM2 antagonist **SAR405838** were developed. The results have shown that in both, the in vitro and in vivo models of acute leukemia, there is no cross-resistance to BCL-2 and HDM2 inhibitors. The combination of ABT-263 and **SAR405838**, both at 50 mg/kg, resulted in complete tumor regression in 100% of mice for a period of 44 days, and this treatment regime was much more effective than the single agent therapy [10].

#### 4.3.2. Agents Downregulating the Level of Anti-Apoptotic BCL-2 Family Proteins

In addition to the compounds which directly bind BCL-2, combinations with agents that indirectly downregulate the level of anti-apoptotic BCL-2 have been also reported. Nilotinib (AMN107, Tasigna), a Bcr-Abl tyrosine kinase antagonist, inhibits the expression of anti-apoptotic BCL-2 proteins [153,154]. In the study designed by Carter et al., **nutlin-3** sensitized the patients’ chronic myeloid leukemia cells to nilotinib treatment, even though the cells were initially resistant to this drug [61]. Oridonin, a bioactive constituent of a Chinese medicinal herb, which previously had been known as a modulator of BCL-2 levels in melanoma and leukemia, is a potent agent in combination therapy [155,156]. **Nutlin-3**/oridonin treatment leads to robust apoptosis through a substantial increase in the proapoptotic/antiapoptotic BCL-2 proteins ratio in p53^wt^ osteosarcoma cells. Moreover, even though oridonin did not increase the level of p53, HDM2, and Puma in the combined strategy **nutlin-3** completely abolished the viability of cancer cells. It has been revealed that the synergistic pro-apoptotic effect occurs due to the upregulation of **nutlin-3**-triggered caspase activation and PARP cleavage [63].

#### 4.3.3. SMAC Mimetic Compounds

SMAC (second mitochondria-derived activator of caspase) is a mitochondrial protein that promotes cytochrome c-dependent caspase activation [157]. The mechanism of SMAC action is to promote apoptosis due to the binding to the inhibitor of apoptotic proteins, IAPs, and blocking their inhibitory activity. Thus, SMAC proteins promote the caspase activation in the cytochrome c/Apaf-1/caspase-9 pathway (Figure 4) [157]. So far, several human IAPs have been identified and one of the representatives is known as the protein XIAP (X-linked inhibitor of apoptosis protein) [158]. 

It has been shown that the level of XIAP is regulated by HDM2 [159]. The hypothesis that the combination of XIAP inhibitors and HDM2 antagonists may lead to the synergistic proapoptotic effects in cancer cells has been verified. The study by Zheng and co-workers has demonstrated that the small molecule XIAP inhibitor, SM-164, does not significantly enhance the potency of **MI-219** against lung cancer cells [47]. On the contrary, another group has claimed that the concomitant inhibition of XIAP and p53 activation promotes apoptosis in blasts from the patients with primary AML. The mechanism of the synergy between **nutlin-3a** and ABT-10 (SMAC mimetic), and **nutlin-3a** and 1396-11 compound (XIAP inhibitor) has been examined. The researches have revealed that the treatment with either combination, ABT-10/**nutlin-3a**, and 1396-11/**nutlin-3a**, induced greater activation of apoptosis signaling pathways and more strongly decreases p21 level via the caspase cleavage, as a result of XIAP inhibition [66]. Also, the cytosolic release of SMAC and increase of effector caspase-6 following the inhibition of HDM2 were found to be responsible for the increased cell sensitivity to XIAP inhibition [66].

#### 4.3.4. TRAIL Agonists

TRAIL (TNF-related apoptosis-inducing ligand) is a member of the TNF (tumor necrosis factor) family of cytokines, which acts as one of the most principal extracellular activators of apoptosis [160,161,162]. TRAIL ligand has an affinity to five receptors, among which DR4 (TRAIL-R1) and DR5 (TRAIL-R2) are able to transmit the apoptotic signal [161,163]. The binding of TRAIL to the death receptors DR4 and DR5 engages death-inducing signaling complex, DISC with Fas-associated death domain, FADD and induces cleavage of caspase 8. As a consequence, the process leads to the activation of caspase 3 or the engagement of the mitochondrial apoptotic pathway (Figure 4) [164,165]. 

Death receptors activate apoptosis pathway in vitro and in vivo after binding the recombinant human TRAIL (rhTRAIL) [166,167,168,169]. rhTRAIL induces selective apoptosis in cancer cells and leaves the normal cells unharmed [170,171].

The synergistic interactions of both **nutlin-3** and rhTRAIL (dularemin) and **nutlin-3** and DR-5-selective TRAIL variant, D269H/E195R [172], have been estimated by the researchers in Groningen [67]. The synergistic proapoptotic effect, induced by enhanced caspase 3 cleavage, has been observed for both combinations. The study has shown that nutlin-3 increases the expression of DR5 on the cell surface in p53^wt^ human non-small cell lung carcinoma, ovarian cancer, and colon cancer cell lines. Relating to this fact, the combination of **nutlin-3** with D269H/E195R has been more effective in inducing apoptosis than **nutlin-3**/rhTRAIL treatment. Furthermore, the **nutlin-3**/TRAIL agonist/cisplatin triple combination treatment has been explored in the ex vivo tissue slice model of the primary human ovarian cancer. Adding cisplatin as a genotoxic agent to the combination intensified DR5-mediated apoptosis [67]. 

The combined targeting of HDM2 and TRAIL has also been reported in another study. Urso and colleagues have investigated the synergy between **nutlin-3** and rhTRAIL in malignant pleural mesothelioma (MPM) cell lines and **RG7112** and rhTRAIL in the in vivo MPM mouse model [68]. The authors speculated that the synergy between **nutlin-3a** and TRAIL results from the engagement of mitochondrial apoptosis by TRAIL, accompanied by the increased DR5 expression and decreased survivin expression in a response to HDM2 antagonist [68].

### 4.4. Combination with Therapy Selectively Targetting Pro-Survival Signaling Pathways

As our knowledge of the molecular basis of carcinogenesis increases, so do the possibilities of designing new therapies for eradicating the disease. We now know that in cancer cells aberrations occur in many cellular signaling pathways. These pathways regulate, among other processes, survival, growth, and proliferation [173]. The approach to cancer treatment aiming at the selective targeting of these pathways, designated as the targeted therapy, has gained considerable acceptance in the past 20 years. Throughout these years, targeted monoclonal antibodies and synthetic small molecule drugs were extensively tested, in many cases providing much more precise tools than chemotherapy, radiotherapy or other conventional treatments [173,174,175].

**Figure 5:**
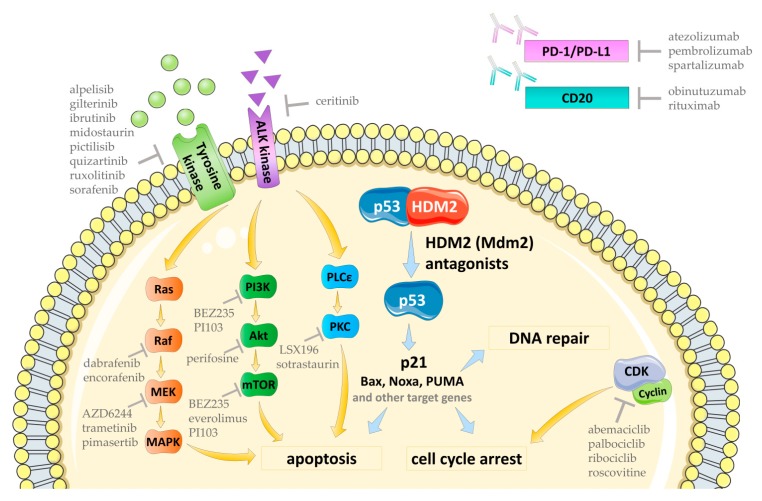
The outline of the combinations of HDM2 antagonists with targeted therapy agents.

Despite the wide range of anticancer drugs for targeted therapy and many successes in the preclinical research, a great percentage of therapeutics does not provide satisfactory therapeutic outcome when delivered to the patients as single agents [176]. Due to this fact, using two or more agents at the same time as combination therapy became the standard of clinical practice. This also accounts for HDM2 antagonists, since over-activated pro-survival pathways in cancer cells greatly antagonize anti-cancer properties of p53 activation. For this reason, inhibition of pro-survival signaling in cancer cells provides a strong synergism with p53-activating compounds (Figure 5).

#### 4.4.1. Inhibitors of Tyrosine Kinases

Tyrosine kinases transfer a phosphate from ATP to tyrosine residues in polypeptides. They function either as extracellular receptors with distinct domains responsible for ligand-binding and kinase activity, or non-receptor proteins located in the cytoplasm [177]. Tyrosine kinase receptors are responsible for initiation, transmission, and regulation of many cellular pathways connected to cell proliferation and survival, such as JAK/STAT, PI3K/Akt/mTOR, and Ras/Raf/MEK/MAPK [177,178]. Tyrosine kinase’s functions are tightly controlled, but due to mutations in cancer cells frequently their constant activation occurs, providing an unrestrained flow of survival signaling [179].

Many tyrosine kinase inhibitors are currently tested in clinical trials, including several combinations with HDM2 antagonists. FLT3 tyrosine kinase inhibitor, midostaurin (PKC412), is tested in phase 1 and 2 clinical study for acute myeloid leukemia in combination with **HDM-201** (NCT03760445). One of the HDM2 antagonists, **DS-3032b**, is under evaluation in phase 1 clinical trial in combination with quizartinib (NCT03552029). This study also featured AML patients, but only with FLT3 mutated cancers. An additional phase 2a/2b clinical study, currently open for recruitment, focuses on combining **KRT232** with ruxolitinib, another inhibitor of tyrosine kinase receptor, which causes an indirect inhibition of JAK/STAT signaling (NCT03669965).

Ibrutinib is a novel inhibitor of Bruton’s tyrosine kinase present on B-cells [180]. This inhibitor has been used in combination with **nutlin-3** and has exerted a synergistic anti-cancer effect on p53^wt^ and p53^mut^ B-leukemic cells [72]. This p53-independent mechanism might be mediated by other proteins from the p53 family, such as p63 and p73 [181]. This effect occurred mainly due to apoptosis induction and cell cycle arrest. When **nutlin-3** was combined with sorafenib, a multi-kinase inhibitor, a synergistic cytotoxic effect was observed in AML cell lines [74]. Apart from the common occurrence of apoptosis induction, Zauli et al. also observed an increase in autophagic cell death upon the dual treatment [74]. Sorafenib was also used in combination with **nutlin-3** in another study, which tested the co-treatment in renal cell carcinoma models [75]. This treatment resulted in increased apoptosis that was attributed to the increased levels of proapoptotic PUMA protein when the sorafenib and **nutlin-3** cotreatment was applied [75]. Midostaurin, was shown to synergize with **CGM097** in AML cell models along with apoptosis induction [51,73]. A more selective inhibitor of FLT3, quizartinib (ACC220), also showed similar synergy [73]. **CGM097** and **HDM-201** in combination with FLT3 inhibitors, midostaurin, BKM120 (buparlisib), GDC-0941 (pictilisib), BYL719 (alpelisib) and ASP2215 (gilterinib) also exhibited a moderate to strong synergistic response in AML cell lines [44]. Simultaneous activation of p53 by HDM2 blockade and ALK (anaplastic lymphoma kinase) inhibition worked synergistically in suppressing the growth of different ALK-amplified or mutated neuroblastoma cell lines [69]. The researchers used **CGM097** and ceritinib, a second generation ALK inhibitor first in in vitro studies, which were then followed by mouse xenograft experiments. The combination was effective and resulted in tumor regression throughout the time of the treatment in three out of five models used. The authors point out that HDM2 inhibition is especially valuable for the treatment of MYCN-amplified neuroblastoma cell lines, as these cells show remarkable sensitivity to MDM2 antagonists in vitro [69].

#### 4.4.2. Ras/Raf/MEK/MAPK Pathway Inhibitors

One of the best characterized cellular pathways in cell biology is the mitogen-activated protein kinase pathway, composed of Ras/Raf/MEK/MAPK proteins. This pathway is responsible for many critical functions of the cell response to extracellular stimuli, such as cytokines, growth factors or mitogen binding to their receptors localized in the cell membrane [182,183]. The components of this pathway play a very important role in the genesis of cancer, which has been reported many times before [183,184,185]. p53 and the MAPK components are the most frequently mutated tumor suppressor and oncogene pathways in human cancers [81]. For this reason, there is a great potential in simultaneous targeting of these two pathways to provide useful therapeutic approaches. For example, MEK inhibition seems to result in transcriptional and posttranscriptional downregulation of the *HDM2* gene, which in turn leads to lower HDM2 protein levels and increased activity of p53 [186].

The inhibitors of the Ras/Raf/MEK/MAPK pathway are used in combination with various HDM2 inhibitors in four clinical trials, one of which is still recruiting. **AMG-232** was tested in combination with trametinib (GSK1120212), a selective MEK1 and MEK2 inhibitor in a phase 1b clinical trial on the AML patients (NCT02016729). Another clinical study (NCT02110355) featured **AMG-232** for the treatment of metastatic melanoma along with trametinib and dabrafenib. The early data for **AMG-232** combined with trametinib or dabrafenib suggested promising pharmacokinetic properties and potential antitumor activity [78]. **HDM-201** has also been combined with trametinib in a clinical study for the treatment of advanced/metastatic RAS/RAF mutant and p53^wt^ colorectal carcinomas (NCT03714958). This phase 1 study sponsored by Centre Léon Bérard is currently recruiting and is estimated to be completed in late 2020. A phase 1 clinical trial of yet another HDM2 antagonist, **SAR405838** in combination with pimasertib, a selective MEK1/2 inhibitor, has been conducted for the treatment of malignant neoplasm (NCT01985191). The patients suffered from different tumor types, i.e., colorectal, lung, melanoma. The trial has so far defined the maximum tolerated dose of the drugs in combination and provided preliminary results on the antitumor activity of this combination [81].

Recently, Wu and co-workers confirmed the synergistic effect of trametinib with three different HDM2/p53 interaction inhibitors—**nutlin-3**, **idasanutlin**, and **HDM-201**—in cutaneous melanoma cell models [82]. All combinations resulted in a significant increase of p53 level as well as its phosphorylation status. The authors propose that the synergy results from the sustained activation of ATM following trametinib administration, which maintains high phosphorylation status and activity of p53, released by HDM2 antagonists. As a result, increased levels of apoptotic and cell-cycle arrested p53^wt^ cells (but not p53^mut^) were also reported, showing a clear synergy of trametinib with HDM2 antagonists [82]. A significant synergistic effect of combined HDM2/p53 and MEK blockade on cell growth inhibition and apoptosis induction was observed by Zhang et al. in AML cell lines [77]. This group used **nutlin-3a** and a second generation allosteric MEK1/2 inhibitor AZD6244 (AstraZeneca, ARRY-142886, selumetinib), which showed high selectivity and anti-tumor efficiency both in pre-clinical and clinical studies [187]. The MEK inhibitor itself has shown moderate proapoptotic effect in OCI/AML3 and MOLM13 cells, which became significant upon combining selumetinib with **nutlin-3a** [77]. **CGM097** has been tested for synergy in combination with LGX818 (NVP-LGX818, encorafenib), a BRAF inhibitor [80]. The authors reported a strong synergistic response to combination treatment in both the in vitro viability assays on BRAF^mut^ melanoma cells, and tumor growth in vivo, to the point of long-lasting tumor regressions. The proposed rationale for this synergism was the induction of a complementary set of anti-proliferative and apoptosis stimulating molecules by the combination of the compounds, since **CGM097** induces p21 and Bax expression, while NVP-LGX818 causes the induction of p27 and Bim [80]. **CGM097** seems to exhibit a synergistic apoptotic effect also when combined with selumetinib [73].

#### 4.4.3. PI3K/Akt/mTOR Pathway Inhibitors

PI3K/Akt/mTOR pathway has very important functions in controlling various cellular processes. Among them are transcription and translation, metabolism, angiogenesis, cell cycle and apoptosis [188,189]. These processes are important both in physiological and in pathological conditions and are very often deregulated during cancer formation and progression [190]. 

Perifosine, a synthetic alkyl-phospholipid, which is known to inhibit Akt, has been combined with **nutlin-3** and showed increased antileukemic activity [86,191]. The combination has been tested on six different leukemia cell lines and resulted in greater apoptosis and p53 protein expression induction in p53^wt^ cell lines [86]. **Idasanutlin** and BEZ235, a PI3K and mTOR inhibitor, elicited a strong therapeutic response in well-differentiated/dedifferentiated liposarcomas, which corresponded with consistent induction of apoptosis and p53 level upon the combined treatment [83]. These findings were then confirmed on mouse xenograft models, in which the dual therapy caused significant tumor regression [83]. A different research group has found that PI3K/mTOR inhibitor PI103 causes a reduction in p53 level, which has been raised when a combination with **nutlin-3** was applied [85]. Synergistic effects of HDM2 antagonist **AMG-232** with many PI3K/Akt/mTOR and Ras/Raf/MEK/MAPK inhibitors were also reported on twenty different human cancer cell lines and in RKO tumor xenograft in vivo experiments [79]. A great therapeutic effect was observed upon combining HDM2 antagonist **AMG-232** with MEK inhibitor PD0325901 and PI3K inhibitor AMG511 in triple therapy [79]. Dual therapy with **CGM097** and everolimus, a well-known mTOR inhibitor, showed an additive antiproliferative effect on GOT1 cells [32].

#### 4.4.4. CDK Inhibitors

Alterations in cell cycle control occur frequently in many types of human cancers. For this reason, inhibitors of cyclin-dependent kinases (CDKs) became a very important group of anti-cancer drugs [192]. In cancer cells, CDK regulatory pathways are constantly active, leading to the omission of critical cell cycle checkpoints, uncontrolled growth, and proliferation [192].

One combination therapy using an HDM2/p53 interaction inhibitor and a CDK inhibitor has made it into the clinical trials. **HDM-201** has been combined with a selective CDK4/6 inhibitor ribociclib (LEE011) from Novartis for the treatment of 74 adults with liposarcomas (NCT02343172). This Phase 1b/2 study aims to assess the maximum tolerated dose and the safety and efficacy of the combination for liposarcoma patients. 

The possibilities of combining the inhibitors targeting HDM2 and CDKs are being continuously explored in preclinical research. The combination of **idasanutlin** with palbociclib, a highly selective inhibitor of CDK4/6 widely used in clinical trials, was reported to wield great antitumor properties in dedifferentiated liposarcomas when compared to monotherapy [88]. Simultaneous treatment caused a significant increase in apoptosis in the in vitro studies, which was later confirmed by tumor volume regression in the in vivo experiments with a mice xenograft model of dedifferentiated liposarcoma [88]. However, Sriraman et al. showed that in five different cell lines derived from human liposarcoma, HDM2 inhibitor **nutlin-3a** and CDK4/6 inhibitors palbociclib, ribociclib and abemaciclib not only fail to synergize, but even seem to antagonize each other’s therapeutic effects, despite the co-amplification of HDM2 and CDK4 genes in the used models [87]. The authors concluded that employing both therapies simultaneously may, in fact, be counterproductive and result in an unfavorable outcome for patients [87]. These findings seem to contradict the previous report by Laroche-Clary et al. [88]. Roscovitine (CY-202, seliciclib), a wide-range CDK inhibitor with strong activity towards CDK1, CDK2, CDK5, and CDK7, and weak towards CDK4 and CDK6, showed a considerable synergy with **nutlin-3a** [90,193]. The effect was visible as an additive reduction of cell viability and induction of p53-dependent apoptosis. The same researchers reported that the effect was enhanced when combining **nutlin-3a** and roscovitine with DRB, an adenosine analog [90]. Roscovitine and **nutlin-3** were additionally found to pharmacologically synergize in neuroblastoma [89].

#### 4.4.5. PKC Inhibitors

Another class of protein kinases extensively studied in the context of cancer is the family of Protein Kinases C (PKC), composed of at least twelve members [194,195]. Different isoenzymes of PKC affect many cell functions, such as proliferation, migration, adhesion, survival and malignant transformation, and are crucial players in carcinogenesis and maintenance of malignant phenotype of cancer cells [194,196]. Overexpression of PKCs is often observed in cancer [194,197].

A therapeutic combination of LXS196, a small-molecule drug that binds and inhibits PKC, and **HDM-201** is being determined in the form of an active clinical trial in metastatic uveal melanoma patients (NCT02601378). The combination of PKC inhibitor and HDM2 antagonists has been tested for uveal melanoma also in nonclinical research. Co-treatment using **CGM097** and a PKC inhibitor sotrastaurin (AEB071) was reported by Carita et al. to decrease the tumor volume in patient-derived xenograft mouse models [91]. The same tendency to stimulate cell death by apoptosis has also been confirmed in uveal melanoma cell lines [91].

### 4.5. The Combination with Anti-PD-/PD-L1 and Anti-CD20 Therapeutic Antibodies

Monoclonal antibodies (mAbs) constitute a tremendous portion of anti-cancer agents. Targeted therapies based on monoclonal antibodies that block numerous oncogenic pathways are widely studied [198,199]. mAbs are the most successful therapeutic agents, for both, the solid tumors [200], and leukemias or lymphomas [201]. The unprecedented potency of immunoglobulins has been confirmed by hundreds of clinical trials (according to the 2017 GlobalData report [202]).

A number of combinations of HDM2 antagonists with monoclonal antibodies are undergoing clinical trials. Among them, the therapies with mAbs targeting PD-1/PD-L1 interaction or B-lymphocyte antigen CD20 have their primacy (Figure 5).

In June 2019 there have already been three 1b/2 phase clinical studies regarding the combination of HDM2 with PD-1 or PD-L1 inhibitors. In 2016, Novartis Pharmaceuticals has started clinical trials with the combination of **HDM-201** and PDR001 (spartalizumab, anti-PD-1 antibody) to characterize the dose escalation for colorectal cancer and renal cell carcinoma treatment (NCT02890069). Pembrolizumab (Keytruda), another anti-PD-1 antibody approved by the FDA across a range of cancers [203] is tested with **APG-115** in patients with advanced solid tumors and unresectable or metastatic melanomas (NCT03611868). Another active clinical trial involves the studies on the safety and tolerability of **idasanutlin** in combination with atezolizumab (Tecentriq, anti-PD-L1 antibody, a drug approved as a single therapy for various cancers [203]) and MEK inhibitor, cobimetinib. The study has been running since 2018 and is currently in the recruitment phase for the patients who suffer from metastatic estrogen receptor-positive breast cancer (NCT03566485).

Rituximab (MabThera^®^) and obinutuzumab (Gazyva^®^) are humanized anti-CD20 mAbs characterized by a distinct mechanism of action. The scientists speculate on the superiority of these two biologics over each other [204]. Pre-clinical data suggests that obinutuzumab is more efficient than rituximab in depleting B cells [205] and has a better toxicological profile, while rituximab therapy causes increased resistance generation [206,207]. Nevertheless, both mAbs have been approved for cancer therapy.

Phase 1b/2 clinical trials of rituximab and obinutuzumab in combination with **idasanutlin** have been conducted by Hoffmann-La Roche. The first study started in 2015 and involved the treatment with **idasanutlin** and obinutuzumab in the FL patients and the **idasanutlin**/rituximab combination in DLBCL participants (NCT02624986). The second study is running and aims at determining the regimen for **idasanutlin** and venetoclax (selective BCL-2 protein inhibitor, see the previous section) in dual and triple combinations with obinutuzumab and with rituximab for FL and DLBCL patients (NCT03135262). 

In addition, the synergistic effect on cell death induction has been shown for obinutuzumab and **nutlin-3** in the preliminary in vitro studies with chronic lymphocytic leukemia cell lines [92]. Roche has strengthened the idea of combination therapy with anti-CD20 mAbs and HDM2 antagonist for the patients with CD20-positive B-cell malignancies. The study reported the combined obinutuzumab/**idasanutlin** treatment to be superior to single therapy with mAb in mantle cell lymphoma (MCL) and DLBCL xenograft models [93].

The efficacy of drozitumab, a human mAb against DR5 was evaluated in a combination treatment with **nutlin-3**. HDM2 antagonist has augmented the apoptotic response to drozitumab in vitro in a panel of sarcoma cell lines and ex vivo human sarcoma patients. The scientists have postulated that the key factor that contributes to the synergistic effects between these molecules is upregulation of DR5 receptor expression following the p53 activation [94]. However, the development of drozitumab has been discontinued in 2010 [208]. 

### 4.6. The Combinations with Miscellaneous Anti-Cancer Agents

#### 4.6.1. Proteasome Inhibitors

Proteasome serves as the main center of protein degradation in cells. The proteasome-dependent degradation of tumor suppressors, such as p53 [188,190], and other proteins involved in anti-cancer response (i.e., IκB and p44/42 MAPK) [209] is correlated with multiple types of cancer [210]. Therefore, inhibition of proteasome activity is a promising anti-cancer strategy (briefly reviewed in [210,211]). 

Bortezomib (Velcade^®^) was the first proteasome inhibitor designed and approved for cancer treatment, more specifically for multiple myeloma patients [212]. The synergistic effect of bortezomib in combination with HDM2 inhibitors has been evaluated on a variety of cancer types in vitro. Bortezomib has exhibited anti-cancer activity against mantle cell lymphoma, and the addition of **nutlin-3** has resulted in synergistic cytotoxicity regardless of mutational p53 status in cancer cell lines [97]. The p53-independent effect of the combination has been associated with an increased level of p73 protein, a p53 homolog. p73-mediated induction of p21, Puma and Noxa expression may account for the observed cell-cycle arrest and apoptosis [97]. An additive cytotoxic effect of **nutlin-3** and bortezomib has been reported on bortezomib-sensitive multiple myeloma (MM) cell lines [95]. The authors explain that the combination of HDM2 inhibition and proteasome inhibition not only increases the stability and levels of p53 but also blocks its ubiquitination and transcriptional inactivation, thus making p53 more functional [95]. In another study, the scientists have shown the synergistic cytotoxic activity of the compounds in MM cells. The authors report the induction of apoptotic signaling defined as the cleavage of PARP, caspase-8 and caspase-3 and a modest cleavage of caspase-9 [96]. Additionally, p53-induced repression of the anti-apoptotic protein, survivin was observed [96]. In breast, prostate, colon, and thyroid cell lines, the **nutlin-3**/bortezomib combination has triggered the synergistic cytotoxic effect. Furthermore, the scientists have postulated that, as a consequence of **nutlin-3** action, molecular pathways associated with poorer responsiveness to bortezomib may be counteracted [95].

Ixazomib citrate and carfilzomib are next-generation proteasome inhibitors, that are used in experimental combination therapies with HDM2 antagonists. Ixazomib citrate is currently tested in a combination with **idasanutlin** and dexamethasone. Phase 1 and 2 clinical study is performed to estimate optimum regimens in treating patients with MM that has returned after a period of improvement (NCT02633059). National Cancer Institute conducts the phase 1 trial studies on **AMG-232** treatment when given together with carfilzomib and other drugs used in chemotherapy: lenalidomide and dexamethasone. The patients suffering from hypercalcemia, plasmacytoma, recurrent plasma cell myeloma, and refractory cell myeloma are currently being recruited (NCT03031730).

The effect of MG-132 (one of the early proteasome inhibitors) and **nutlin-3** on schwannoma cells has been assessed by Chen and colleagues. They have demonstrated that the differences in the expression of merlin, which is a tumor suppressor protein, in cancer cultures explain the functional diversity in response to **nutlin-3** treatment [98]. It has been proved that dual therapy of **nutlin-3**/MG-132 narrows the differences in the sensitivity to **nutlin-3** in schwannoma cells. In addition, in vivo experiment with human xenograft model of schwannoma has revealed that the small-molecules work together to impose apoptosis [98].

#### 4.6.2. Histone Deacetylase Inhibitors

Epigenetic regulation is also used as a possible therapeutic approach for cancer treatment. In this therapy, histone deacetylases (HDAC) inhibitors play a major role, with several potent compounds being tested in clinical trials [213]. Valproic acid (VPA) inhibits the action of class I HDACs [214]. The combination with **nutlin-3** increased the level of apoptosis and autophagy in p53^wt^ AML cell line MOLM-13 and caused significant tumor regression in a MOLM-13 mouse model [100]. Suberoylanilide hydroxamic acid (SAHA, vorinostat, MK0683) is another HDAC inhibitor. It has been used in combined therapy with **idasanutlin**, resulting in synergy in apoptosis induction in AML cell lines regardless of their p53 status [99]. 

#### 4.6.3. Antibiotics

Antibiotics are typically used to treat bacterial infections. However, some classes of antibiotics inhibit the division of proliferating eukaryotic cells and thus are also explored in the therapy of cancer [215,216,217]. Some compounds representative for this group of molecules were studied in combination therapies with HDM2 antagonists. One example is actinomycin D, a well-known transcription inhibitor widely used to treat cancer [218]. Actinomycin D inhibits RNA synthesis by blocking DNA-dependent RNA polymerase. **Nutlin-3** combined with actinomycin D resulted in the enhancement of antitumor activity in rhabdomyosarcoma cells with wild-type p53 [101]. In another study with the same combination, the synergic effect on cancer cell lines has been confirmed [102]. **Nutlin-3a** binds to HDM2 and releases p53, while actinomycin D stimulates p53 phosphorylation and accumulation via a mechanism which engages HDM2 high expression. Authors have shown that the combination therapy results in the enhanced p53 activation with phosphorylation of Ser46 residue, which is a marker of proapoptotic p53_KILLER_ mode [102].

#### 4.6.4. Zinc

Zinc has an essential impact on p53. The p53 protein binds to DNA by a domain which is stabilized by a zinc atom, which is why its role is so important in forming p53 DNA-binding domain complex [219]. Metal chelators have the ability to remove zinc from p53, what in turn leads to ‘mutant-like’ p53 form which cannot specifically bind to DNA [220]. Zinc has been tested in combination with HDM2 inhibitor, **MI-219**, in colon and breast cancer cells. The scientists observed a significant increase of **MI-219**-induced apoptosis in the treated cells. Furthermore, this phenomenon was reversible by a zinc chelator, TPEN (N,N,N′,N′-tetrakis(2-pyridinylmethyl)-1,2-ethanediamine), which clearly suggests a crucial zinc role in this process [38].

Zinc metallochaperones (ZMCs) are a new class of mutant p53 reactivators [221]. In general, the function of metallochaperones is to shuttle metal ions to intracellular proteins [222]. ZMCs are small-molecule metal ion chelators that have the ability to donate zinc to mutant p53 which lacks Zn^2+^ [221]. Many studies indicate that ionizing radiation and chemotherapy work better on wild-type p53 tumors [223]. This became the rationale for combining these therapies with drugs which reactivate p53 in p53-mutated cancers. There have been studies conducted on the combination of **nutlin** with ZMC1 (thiosemicarbazone). The authors hypothesized that if **nutlin** could stabilize the levels of mutant p53, this will result in that ZMC1 should have more mutant p53 to reactivate and in turn kill more cancer cells. The results of this combination therapy on immunodeficient mice bearing TOV112D xenograft tumors have shown a significant synergistic effect on the tumor growth inhibition in comparison to the single treatment [103].

#### 4.6.5. Heat Shock Protein Inhibitor

Heat shock proteins (HSPs) are responsible for the proper folding of synthesized proteins or refolding under denaturating conditions [224]. HSPs have an impact on protein degradation in proteasome [225]. Cancer cells require HSP90 protein for the maintenance of mutated proteins. In patients with acute myeloid leukemia, there is a high expression of HSPs [226]. Inhibitors of Hsp90 increase the acetylation of p53 and inhibit HDMX, indicating the potential synergy with HDM2 antagonists in enhancing p53-mediated apoptosis. Combination treatment of Hsp90 inhibitor geldanamycin and **nutlin-3** showed synergistic effect by inducing apoptosis in AML cell lines and primary AML cells [104]. The results also revealed that **nutlin-3** stimulates acetylation of p53, histones, and heat shock proteins [104].

#### 4.6.6. ATPase Inhibitors 

Archazolid is a myxobacteria-derived ATPase inhibitor. The compound regulates anoikis resistance and exhibits anti-tumor and anti-metastatic activity in hepatocellular carcinoma (HCC) and breast cancer in both in vivo and in vitro models [227,228,229]. Schneider and colleagues have reported that the archazolid/**nutlin-3** combination results in synergistically induced cell death in p53^wt^-harboring cancer cells in vitro and in a glioblastoma xenograft model [105]. The pro-apoptotic effect was related to the activation of Bax and IGFBP3 (insulin-like growth factor-binding protein 3) pathways. It has also been disclosed that the observed synergy might be a consequence of the reduction of glycolysis due to the intensified downregulation of TIGAR (TP53-inducible glycolysis and apoptosis regulator) mRNA level and also diminished Glu1 levels and glucose uptake in cancer cells [105].

#### 4.6.7. Mitotic Inhibitors

Mitotic inhibitors, especially spindle poisons, are claimed to have a relatively low genotoxicity score [230]. Spindle poisons were evaluated in pre-clinical trials as a single treatment, as well as in combination with HDM2/p53 antagonists. Vincristine in combination with **nutlin-3** increased anti-cancer activity in rhabdomyosarcoma (RMS) tumors harboring wild-type p53 [101]. Vincristine was also combined with **RG7112** in infant mixed lineage leukemia-rearranged acute lymphoblastic leukemia (MLL-ALL). There was a therapeutic enhancement in comparison compared to the single therapy with **RG7112** [106]. Paclitaxel, another example of spindle poisons, is currently under evaluation in clinical trials in combination with **ALRN-6924** (NCT03725436). This study is being conducted on patients with advanced, metastatic, or unresectable solid tumors. It is a promising strategy of anti-tumor therapy for patients with tumors which cannot be eliminated by the surgery. 

#### 4.6.8. Other Agents

Interestingly, metformin, a drug widely used in the treatment of type 2 diabetes mellitus and polycystic ovary syndrome, seems to possess anti-tumor properties via indirect mTOR protein inhibition [231,232]. One of the studies focused on combining metformin with **nutlin-3a**, and this dual treatment of p53^wt^ mesothelioma cells showed additive growth inhibition of the cells [107]. A combination of **nutlin-3** and Tanshinone IIA, a phytochemical isolated from the Chinese medicinal herb *Salvia miltiorrhiza*, seems to show a synergistic effect when used together in AML cell lines. The authors reported increased cytotoxicity, apoptosis, autophagic cell death and cell cycle arrest, as well as p53 upregulation and PI3K/Akt/mTOR pathway inhibition in the in vitro leukemia models and cell sampled from patients [108]. Within the antimetabolites group, there are analogs of folic acid such as methotrexate. The compound inhibits key enzymes in nucleotide biosynthesis, leading to cell-cycle arrest and apoptosis. Methotrexate combined with **nutlin-3a** resulted in a decline of cytotoxicity evoked by **nutlin-3** [42]. One additional study has shown that stapled peptide **ATSP-7041** has an additive effect on cytotoxicity and induction of cell death in Ewing's sarcoma cells with USP7 inhibitor P5091 [57].

## 5. Conclusions

Undeniably, p53 is amongst the most important and best-studied proteins in the context of cancer. Since the discovery of the cancer-preventing potential of p53 and its ubiquitous inactivation in all types of cancers, multiple approaches aiming at its reactivation have been proposed [233]. The development of small-molecule HDM2 antagonists has become one of the leading strategies in this field since it was estimated that in around half of the cancers the wild-type status of the *TP53* gene is preserved.

Following the initial discovery of the nutlins, multiple chemical scaffolds were tested for the ability to release p53 from HDM2 inhibition [31]. These extensive studies have produced several extraordinary compounds, with proven bioactivity and optimized pharmacological properties that were applicable for clinical trials. By that time, however, extensive evidence pointing to serious limitations of the anticancer properties of HDM2 antagonists has accumulated, as these compounds force p53 release without providing appropriate proapoptotic context. It seems that this judgment has been well acknowledged for the past years. This is manifested by the extensive investigation of drug combinations designed in order to support the anticancer properties of HDM2 antagonists (Figure 1). Also, this trend has determined the direction of the experimental clinical trials, where multiple combinations are tested in a search for potential therapeutic synergies. It seems only a matter of time that some combinations will confirm their clinical relevance and will constitute new regimes for the treatment of cancer patients.

## Figures and Tables

**Table 1:** The summary of combination therapies of HDM2 antagonists.

Mode of Action		Combination		In Vitro Studies	In Vivo Studies	Clinical Trials	References
		Therapeutic Agent	HDM2 Antagonist				
**DNA Damage**		5-azacitadine	DS-3032b	-	-	AML or MDS, Phase 1	NCT02319369
		5-fluorouracil	CGM097	neuroendocrine tumor cell lines	-	-	[32]
		acadesine	nutlin-3a	CLL	-	-	[33]
		busulfan	idasanutlin	neuroblastoma	-	-	[34]
		carboplatin	AMG-232	different tumor cell lines	osteosarcoma, colorectal carcinoma, melanoma, NSCLC xenograft mouse models	-	[35]
			APG-115	-	-	salivary gland carcinoma, Phase 1/2	NCT03781986
			nutlin-3a	breast cancer	breast cancer xenograft mouse models	-	[36]
		cisplatin	AMG-232	different tumor cell lines	osteosarcoma, colorectal carcinoma, melanoma, NSCLC xenograft mouse models	-	[35]
			idasanutlin	ovarian cancer, neuroblastoma	-	-	[34,37]
			MI-319	pancreatic cancer	-	-	[38]
			nutlin-3	nasopharyngeal carcinoma, adenocarcinoma, ovarian cancer	-	-	[37,39,40,41]
			nutlin-3a	liposarcoma, RMS, neuroblastoma, osteosarcoma	-	-	[42,43]
		chlorambucil	nutlin-3a	CLL	-	-	[33]
		CPT-11	AMG-232	different tumor cell lines	osteosarcoma, colorectal carcinoma, melanoma, NSCLC xenograft mouse models	-	[35]
		cytarabine	ALRN-6924	-	-	AML and AMS, Phase 1	NCT02909972
			CGM097	AML	-	-	[44]
			HDM-201	AML	-	AML, Phase 1/2	[44] NCT03760445
			idasanutlin	-	-	AML, Phase 1/2, Phase 3	NCT03850535 NCT02545283
			RG7112	-	-	AML, Phase 1	NCT01635296
		daunorubicin	HDM-201	-	-	AML, Phase 1/2	NCT03760445
			idasanutlin	-	-	AML, Phase 1/2	NCT03850535
**DNA Damage**		decitabine	AMG-232	-	-	AML, Phase 1	NCT03041688
		doxorubicin	AMG-232	different tumor cell lines	osteosarcoma, colorectal carcinoma, melanoma, NSCLC xenograft mouse models	-	[35]
			idasanutlin	neuroblastoma	-	-	[34]
			nutlin-3a	DLBCL, CLL, PDAC, liposarcoma, RMS and osteosarcoma	DLBCL xenograft mouse models	-	[33,42,45,46]
		etoposide	MI-219	lung cancer	-	-	[47]
			nutlin-3	ovarian cancer	-	-	[41]
			nutlin-3a	PDAC, neuroblastoma	-	-	[43,46]
		fludarabine	nutlin-3a	CLL	-	-	[33,48]
		gemcitabine	MI-63	MCL	-	-	[49]
			nutlin-3a	PDAC	-	-	[46]
		idarubicin	CGM097	AML	-	-	[44]
			HDM-201				
		ionizing radiation (IR)	AMG-232	-	adenoid cystic carcinoma xenograft mouse models	glioblastoma, Phase 1, soft tissue sarcoma, Phase 1	[50] NCT03107780 NCT03217266
			RG7388	-	RMS xenograft mouse models	-	[51]
			nutlin-3	LSCC, glioblastoma	-	-	[52,53]
		oxaliplatin	MI-219	PDAC, colon and breast cancer	-	-	[54]
		temozolomide	CGM097	neuroendocrine tumor cell lines	-	-	[32]
			idasanutlin	neuroblastoma	-	-	[34]
			RG7775	neuroblastoma	neuroblastoma mouse xenograft models	-	[55]
		topotecan	idasanutlin	neuroblastoma	-	-	[34]
		trabectedin	RG7112	fibrosarcoma and liposarcoma	-	-	[56]
**DNA Damage Response Sustaining**		GSK2830371	ATSP-7041	Ewing’s sarcoma	-	-	[57]
			HDM-201	human melanoma	-	-	[15]
			idasanutlin				
			nutlin-3				
			nutlin-3a	osteosarcoma, breast, colon adenocarcinoma	-	-	[58]
		rucaparib	idasanutlin	ovarian cancer	-	-	[59]
			nutlin-3				
**Apoptosis Induction**	**BCL-2 Inhibitors**	ABT-263	SAR405838	ALL and AML	ALL and AML xenograft mouse models	-	[10]
		ABT-737	MI-63	multiple myeloma	multiple myeloma xenograft mouse models	-	[60,61,62]
			nutlin-3	AML, CML	-	-	[60,61]
	**BCL-2 Dowregulators**	oridonin	nutlin-3	osteosarcoma	-	-	[63]
		venetoclax	idasanutlin	AML, neuroblastoma	AML, neuroblastoma xenograft mouse models	AML patients, Phase 1b/2	[64,65] NCT02670044
		venetoclax + obinutuzumab or rituximab	idasanutlin	-	-	follicular lymphoma and DLBCL	NCT03135262
	**SMAC Mimetics**	1396-11	nutlin-3a	AML	-	-	[66]
		ABT-10					
		SM-164	MI-219	lung cancer	-	-	[47]
	**TRAIL Agonists**	D269H/E195R	nutlin-3	NSCLC, colon, ovarian cancer	-	-	[67]
		rhTRAIL	nutlin-3	NSCLC, colon, ovarian cancer	-	-	[67]
			nutlin-3a	MPM	-	-	[68]
			RG7112	-	MPM xenograft mouse models	-	[68]
**Pro-survival Signaling Pathways Targeting**	**Tyrosine Kinase Inhibitors**	alpelisib	CGM097	AML	-	-	[44]
			HDM-201				
		buparlisib	CGM097	AML	-	-	[44]
			HDM-201				
		ceritinib	CGM097	neuroblastoma	neuroblastoma xenograft mouse models	-	[69]
		dasatinib	nutlin-3	B-CLL	-	-	[70]
			nutlin-3a	ALL	-	-	[71]
		gilterinib	CGM097	AML	-	-	[44]
			HDM-201				
		ibrutinib	nutlin-3	CLL	CLL xenograft mouse models	-	[72]
		imatinib	nutlin-3a	ALL	-	-	[71]
		midostaurin	CGM097	AML	AML xenograft mouse models	-	[44,73]
			HDM-201	AML	-	AML, Phase 1/2	[44] NCT03760445
		nilotinib	nutlin-3	AML, CML	-	-	[60,61]
			nutlin-3a	ALL	-	-	[71]
		pictilisib	CGM097	AML	-	-	[44]
			HDM-201				
**Pro-Survival Signaling Pathways Targeting**	**Tyrosine Kinase Inhibitors**	quizartinib	CGM097	AML	AML xenograft mouse models	-	[73]
			DS-3032b	-	-	AML, Phase 1	NCT03552029
		ruxolitinib	KRT232	-	-	PV, Phase 2a/2b	NCT03669965
		sorafenib	nutlin-3	AML, RCC	-	-	[74,75]
	**Ras/Raf/MEK/MAPK Inhibitors**	AZD6244	CGM097	AML	-	-	[76]
			nutlin-3a	AML	-	-	[77]
		dabrafenib	AMG-232	different human cancer cell lines	colorectal cancer xenograft mouse models	melanoma, Phase 1	NCT02110355 [78,79]
		LGX818	CGM097	melanoma	melanoma mouse models	-	[80]
		PD0325901	AMG-232	different human cancer cell lines	colorectal cancer xenograft mouse models	-	[79]
		pimasertib	SAR405838	-	-	different tumor types, Phase 1	NCT01985191 [81]
		trametinib	AMG-232	different human cancer cell lines	colorectal cancer xenograft mouse models	AML, Phase 1b melanoma, Phase 1	[78,79] NCT02016729 NCT02110355
			HDM-201	cutaneous melanoma	-	colorectal carcinoma, Phase 1	[82] NCT03714958
			idasanutlin	cutaneous melanoma	-	-	[82]
			nutlin-3				
		vemurafenib	AMG-232	different human cancer cell lines	colorectal cancer xenograft mouse models	-	[79]
	**PI3K/Akt/mTOR Inhibitors**	AMG511	AMG-232	different human cancer cell lines	colorectal cancer xenograft mouse models	-	[79]
		AZD8055	AMG-232	different human cancer cell lines	colorectal cancer xenograft mouse models	-	[79]
		BEZ235	AMG-232	different human cancer cell lines	colorectal cancer xenograft mouse models	-	[79]
			idasanutlin	liposarcoma	liposarcoma xenograft mouse models	-	[83]
		everolimus	CGM097	neuroendocrine tumor	-	-	[32]
		GDC-0941	AMG-232	different human cancer cell lines	colorectal cancer xenograft mouse models	-	[79]
		Ly294002	nutlin-3	ALL	-	-	[84]
		MK-2206	AMG-232	different human cancer cell lines	colorectal cancer xenograft mouse models	-	[79]
		PI103	nutlin-3	AML	-	-	[85]
		perifosine	nutlin-3	AML, B-CLL	-	-	[86]
**Pro-Survival Signaling Pathways Targeting**	**CDK Inhibitors**	abemaciclib	nutlin-3a	liposarcoma	-	-	[87]
		palbociclib	idasanutlin	liposarcoma	liposarcoma xenograft mouse models	-	[88]
			nutlin-3a	liposarcoma	-	-	[87]
		ribociclib	HDM-201	-	-	liposarcomas, Phase 1b/2	NCT02343172
			nutlin-3a	liposarcoma	-	-	[87]
		seliciclib	nutlin-3	neuroblastoma	-	-	[89]
			nutlin-3a	melanoma, breast, colon adenocarcinoma, liver carcinoma	-	-	[90]
	**PKC Inhibitors**	LXS196	HDM-201	-	-	metastatic uveal melanoma, Phase 1	NCT02601378
		sotrastaurin	CGM097	uveal melanoma	uveal melanoma xenograft mouse models	-	[91]
**Therapeutic Antibodies**	**Anti-PD-1 and Anti PD-L1**	atezolizumab	idasanutlin	-	-	metastatic ER + breast cancer, Phase 1b/2	NCT03566485
		pembrolizumab	APG-115	-	-	unresectable or metastatic melanoma, advanced solid tumors, Phase 1b/2	NCT03611868
		spartalizumab	HDM-201	-	-	colorectal cancer, RCC Phase 1	NCT02890069
	**Anti-CD20**	obinutuzumab	nutlin-3	CLL	-	-	[92]
			idasanutlin	MCL, DLBCL	MCL, DLBCL mouse xenograft models	FL, DLBCL, Phase 1b/2	[93] NCT02624986 NCT03135262
		rituximab	nutlin-3	CLL	-	-	[92]
			idasanutlin	MCL, DLBCL	MCL, DLBCL mouse xenograft models	FL, DLBCL, Phase 1b/2	[93] NCT02624986 NCT03135262
	**Anti-DR5**	drozitumab	nutlin-3a	osteosarcoma, Ewing’s sarcoma	-	-	[94]
**Miscellaneous Anti-Cancer Agents**	**Proteasome Inhibitors**	bortezomib	nutlin-3	MCL, myeloma, breast, prostate, thyroid, colon cancer	-	-	[95,96,97]
		carfilzomib	AMG-232	-	-	multiple myeloma, Phase 1	NCT03031730
		ixazomib citrate	idasanutlin	-	-	multiple myeloma, Phase 1/2	NCT02633059
		MG-132	nutlin-3	schwannoma	schwannoma xenograft mouse models	-	[98]
	**HDACs** **Inhibitors**	SAHA	idasanutlin	AML	-	-	[99]
		VPA	nutlin-3	AML	AML xenograft mouse models	-	[100]
	**Antibiotics**	actinomycin D	nutlin-3	RMS	-		-	[101]
				different cancer cell lines				[102]
	**Zinc**	zinc	MI-219	colon and breast cancer	-	-	[38]
		ZMC1	nutlin	ovarian, lung, nasopharyngeal cancer	ovarian xenograft mouse models	-	[103]
	**HSP Inhibitor**	geldanamycin	nutlin-3	AML	-	-	[104]
	**ATP-ase Inhibitor**	archazolid	nutlin-3a	liver, cervical, breast cancer, glioblastoma	glioblastoma xenograft models	-	[105]
	**Mitotic Inhibitors**	paclitaxel	ALRN-6924	-	-	advanced, metastatic, or unresectable solid tumors, Phase 1	NCT03725436
		vincristine	nutlin-3	RMS	-	-	[101]
			RG7112	leukemia cell lines	MLL-ALL xenograft mouse models	-	[106]
	**Others**	metformin	nutlin-3a	malignant mesothelioma	-	-	[107]
		methotrexate	nutlin-3a	liposarcoma, RMS and osteosarcoma	-	-	[42]
		tanshinone IIA	nutlin-3	AML	-	-	[108]
		P5091	ATSP-7041	Ewing’s sarcoma	-	-	[57]

ALL—acute lymphocytic leukemia, AML—acute myeloid leukemia, AMS—advanced myelodysplastic syndrome, CLL—chronic lymphoblastic leukemia, CML—chronic myeloid leukemia, DLBCL—diffuse large B-cell lymphoma, FL—follicular lymphoma, LSCC—laryngeal carcinoma, MCL—mantle cell lymphoma, MDS—myelodysplastic syndrome, MLL—mixed lineage leukemia, MPM—malignant pleural mesothelioma, NSCLC—non- small cell lung carcinoma, PDAC—pancreatic ductal adenocarcinoma, PV—polycythemia vera, RCC—renal cell carcinoma, RMS—rhabdomyosarcoma.
